# Characteristic and follow-up of subglottic hemangiomas in Iranian children

**Published:** 2010

**Authors:** Seyed Ahmad Tabatabaii, Ghamartaj Khanbabaii, Ali Reza Khatami, Seyed Ali Sharifnia

**Affiliations:** a Assistant Professor of Pediatrics, Pediatric Pulmonology Fellow, Mofid Children Hospital, Shahid Beheshti Medical University, Tehran, Iran; b Assistant Professor of Radiology, Mofid Children Hospital, Shahid Beheshti Medical University, Tehran, Iran; c Assistant Professor of ENT, Iran Army Medical University, 504 Army Hospital, Tehran, Iran

**Keywords:** Subglotic Hemangioma, Laser Therapy, Tracheostomy, Respiratory Distress, Recurrent Stridor

## Abstract

Subglottic hemangiomas are very rare in compare with cutaneous form but can be life-threatening in the proliferating phase of tumor by airway obstruction. It should be considered in any child with recurrent, persistent and/or progressive, inspiratory or biphasic stridor, respiratory distress and feeding difficulties in the first months of life. It should be confirmed by endobronchoscopic evaluation. Affected infants are most likely to experience symptoms between the ages of 6 and 12 weeks. Infants who admitted and referred to our hospital with recurrent stridor, cough and respiratory distress were reviewed.

Infantile hemangiomas are the most common vascular tumor of the skin and probably 4-5% of infants are involved in it.^1^ The cutaneous hemangiomas are benign and self-limited but when involving the vital organs like spines, central nervous system and eyes may herald some developmental anomalies in growing infant. Subglottic hemangiomas are not common but can be life threatening form especially in the growing and proliferating phase of tumor which can cause airway obstruction. Hoarseness, barking cough, recurrent croup and respiratory distress are the most frequent clinical presentations. In the neonatal period the patients are asymptomatic. By the age of 3 months most of them have respiratory symptoms. Almost 20% of them have some cutaneous hemangiomas in a beard distribution.^2^ The exact pathogenesis of infantile hemongioma is poorly understood^3^ but immunohistochemically, subglottic hemangiomas are different from cutaneous form.^4^

Subglottic hemangiomas should be considered in any child with recurrent, persistent and/or progressive, inspiratory or biphasic stridor, respiratory distress and feeding difficulties in the first months of life and it should be confirmed by endobronchoscopic evaluation.

## Case Report

**Figure 1:**
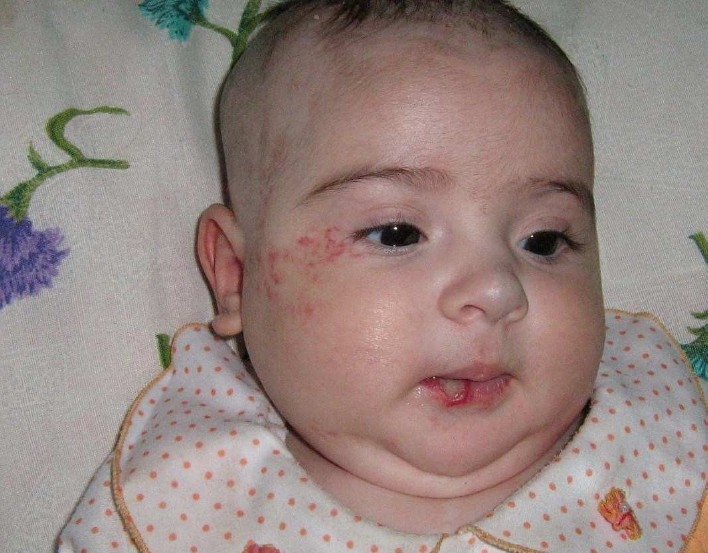
Patient no. 6 (SGH and hemangioma of the face, nose and lip)

Eight infants with subglottic hemangioma are presented follow: they referred from October 2002 to March 2010 to our hospital. The main characteristics are shown in the table 1. All presented with recurrent stridor, cough and respiratory distress with partial response to steroids and bronchodilator. Their age in the first presentation was between one to four months, and five of them were female. Cutaneus hemangioma was noted in most of them. One of them had a large segmental femoral hemangioma, another one had a large segmental hemangioma on the neck and face and four patients had beard distribution (Figure 1). In some of them after a short course of systemic and inhaled steroid therapy a fibroptic bronchoscopic confirmed the diagnosis. Three patients were treated with systemic steroid successfully and now are free of symptoms. The first patient who received KTP laser therapy needed tracheostomy. Decannulation was successful after one week and no further granulation tissue, stenosis or recurrence was noted after a few months of follow-up. For rational management in three of the presented patients who needed tumor removal, elective tracheotomy was performed before laser therapy. Decannulation was successful within 2 weeks. One of these three patients had a severe respiratory distress and needed readmission but bronchoscopic evaluation was normal. She underwent asthma medication with a very good response and after three years, her condition is good. The second patient suffered from atopic disease and had a family history of asthma, she suffered from respiratory distress and generalized wheeze (without stridor or croup) with a very good response to inhaled bronchodilator and steroid.

**Table 1:** Clinical finding and management of eight children with subgllotic hemangiom

No	Sex	Age at first presentation /diagnosis (months)	Symptoms	Associated problems	Medical treatment	Diagnostic method	Percent of obstruction of subglottis	Final treatment	Complication (s) after treatment	Follow up duration/ prognosis
1	F	4/15	Stridor/ wheeze/ RRD*	Asthma- GER**	Oral/aerosol steroid	FOB†	50%	Tracheostomy, KTP†† laser	-	5 years/excellent
2	M	2/11	Stridor/ RRD/ cough	Hemangioma on femur	Steroid	FOB	50%	Tracheostomy KTP laser	Cataract after 4 years	4 years/excellent
3	F	3/4	Stridor/ cough/RRD	Hemangioma on face, lip, gum, ear lid	Oral/aerosol steroid	FOB	75%	Medical treatment	-	3 months/good
4	M	2/4	RRD	GER, anti GER surgery	Oral steroid for 21 month	FOB	30%	Medical treatment	-	7 years/excellent
5	F	3/6	RRD/wheeze/stridor	Ectopia cordis, face hemangioma, asthma	Oral ster-oid/aerosol steroid	FOB	75%	Tracheotomy, KTP	-	3 years/excellent
6	F	1/3	RRD and persistent stridor	Segmental hemangioma on nose, lip & face	Steroid + antibiotic	FOB	75%	Tracheotomy, KTP	-	4 months/good
7	F	1/2	Persistent stridor	Extensive hemangiom on face and neck	Steroid	FOB	75%	Tracheotomy, oral steroid	-	4 months/good
8	F	2/4	RRD and persistent stridor	Extensive hemangiom on face and neck	Steroid Propranolol	FOB	50%	KTP oral steroid Propranolol	-	2 months/good

* RRD: Recurrent Respiratory Distress

** GER: Gastro Esophageal Reflux

† FB: Fiberoptic Bronchoscopy

†† KTP: Potassium-Titanyl-Phosphate

**Figure 2:**
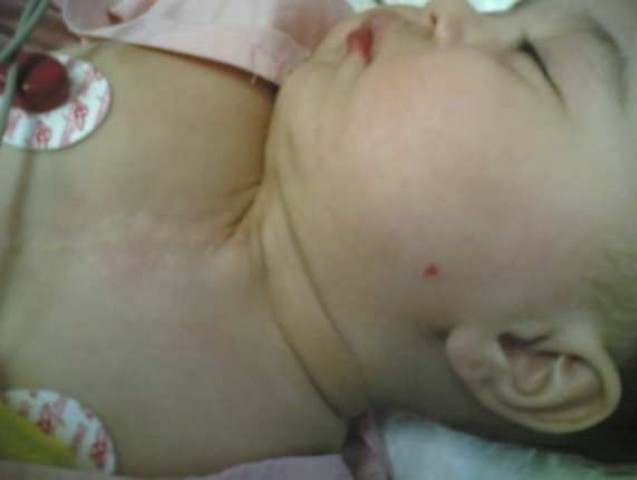
Patient no. 5 (SGH and sternal fissure-corrected at birth and facial hemangioma)

Two had been treated for gastroesophageal reflux without adequate response in respiratory symptoms, and another underwent surgery at birth for ectopic cordis and sternal fissure, and it is considered that she suffered of PHACEs syndrome^5^ (posterior fossa malformation, hemangiomas, arterial anomalies, cardiac defects, eye abnormalities and sternal defect) as only a large segmental hemangioma and one or more anomalies are sufficient for this diagnosis (Figure 2), patient no. 5).

**Figure 3:**
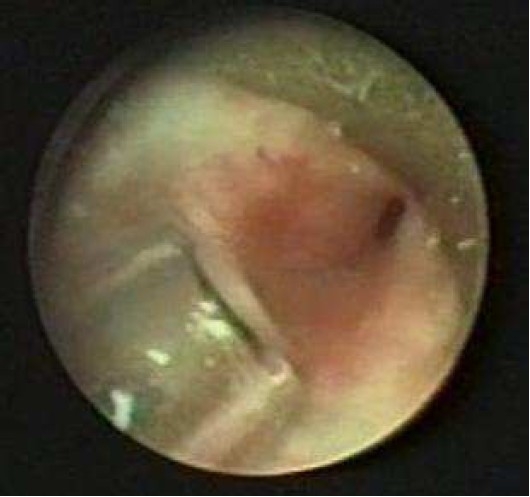
Patient no. 8 before laser therapy

**Figure 4:**
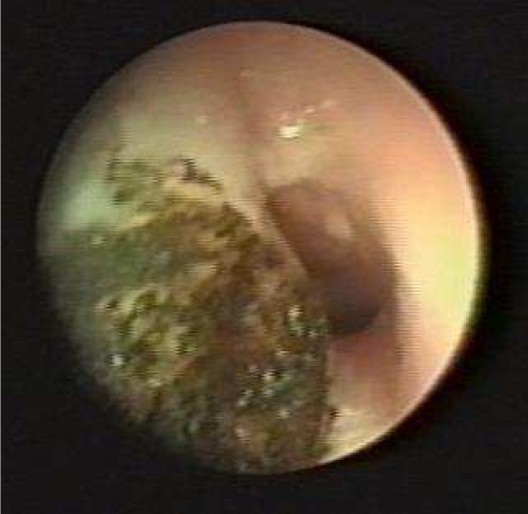
Patient no. 8 during laser therapy

The last patient underwent KTP laser by using jet ventilation and didn’t need tracheotomy (Figure 3 and 4), and now she is taking oral steroid and propranolol.

## Discussion

The present study confirms that when there is inspiratory distress with unknown etiology subglottic hemangioma should be considered. SGH are very rare anomalies and their symptoms are similar with other common respiratory diseases so the diagnosis maybe difficult.^6^ In most patients SGH is present at birth but the diagnosis is late. As in the present study, affected infants are most likely to present symptoms between the ages of 1 and 4 months. Shikhani et al revealed that their symptom will be appear between age of 1 to 6 months because they have a rapid growth during the first year of life.^5^

In the present study the time of diagnosis were 1 to 11 months after presentation of respiratory symptoms. This time is approximately as the same as Shikhani et al study.^5^

The skin hemangiomas may help us to correct diagnosis. In the present study most of patients had cutaneous lesions. In favor of the present study, Orlow et al^2^ found that 63% of their patients who had more than four skin hemangiomas had subglottic hemangioma Sherrington et al^6^ defined that 65% of the patients had skin hemangioma. In a large prospective study^7^ the incidence of PHACE among children with segmental hemangioma was 20%, and 12.5% of the present ones met its criteria. None of those case reports we could find represent the ectopic cordis.^8^

It is known that one of the causes of recurrent or persistent stridor and recurrent respiratory distress may be subglottic hemangioma such as presented patients. Also while Saetti et al^9^ revealed that 30% of the patients with subglottic hemangioma have had stridor and 60% of them presented with signs and symptoms of respiratory distress.

There are various different treatment methods for subglottic hemangiomas but laser therapy is the most common treatment; laser surgery using carbon dioxide was the most common technique for the treatment of subglottic hemangiomas until 2002.^10^

There are some experiences using diode laser, KTP, Nd: YAG.^11^–^13^ KTP was preferred because it has less thermal damage and well absorption by hemoglobin.^14^

Another treatment is corticosteroids therapy.^6^ In the present study systemic steroids are the first choice, which usually has been used before diagnosis due to severe dyspnea. One third of the present cases were treated with steroid alone and now they are symptom free. Sherrington et al revealed that 3% of cases treated only with steroid.^6^ This difference may be due to duration of study or sample size.

The other treatment is propranolol, because propranolol with its vasoconstrictive activity and with decreasing the expression of vascular growth factor and/or triggering apoptosis can decrease the growth of hemangiomas. Leaute-Labreze et al reported 11 infants with capillary hemangiomas. They showed that propranolol can inhibit the growth of capillary hemangiomas.^15^ KTP laserwas used in the present study by using jet ventilation for the last patient, and now she is taking oral steroid and propranolol such as Leaute-Labreze’s study.^15^

Bitar et al^10^ published a systemic review. They showed that the most common used procedures was carbon dioxide, laser surgery, then tracheotomy, systemic corticosteroids, intralesional steroids, and open surgical excision. Also multiple treatments were used in the present study for 75 percent of patients.

## Conclusions

In the present study most of the cases finally needed a multimodality treatment approach including systemic steroid, tracheotomy, and laser ablation. According to our experience the systemic corticosteroid is the first treatment if the lesions are small and the airway is adequate; and multimodality treatment should be considered if the lesions are large and the airway is not adequate.
